# Imbalanced expression of cation-chloride cotransporters as a potential therapeutic target in an Angelman syndrome mouse model

**DOI:** 10.1038/s41598-023-32376-z

**Published:** 2023-04-17

**Authors:** Kiyoshi Egawa, Miho Watanabe, Hideaki Shiraishi, Daisuke Sato, Yukitoshi Takahashi, Saori Nishio, Atsuo Fukuda

**Affiliations:** 1 Department of Pediatrics, Hokkaido University Graduate School of Medicine, Kita 15, Nishi 7, Kita-Ku, Sapporo, 060-8638 Japan; 2 Department of Neurophysiology, Hamamatsu University School of Medicine, 1-20-1 Handayama, Higashi-Ku, Hamamatsu City, Shizuoka 431-3192 Japan; 3 Department of Clinical Research, National Epilepsy Center, NHO, Shizuoka Institute of Epilepsy and Neurological Disorders, Urushiyama 886, Aoi-Ku, Shizuoka, 420-8688 Japan; 4 Department of Rheumatology, Endocrinology, and Nephrology, Hokkaido University Graduate School of Medicine, Kita 15, Nishi 7, Kita-Ku, Sapporo, 060-8638 Japan

**Keywords:** Diseases of the nervous system, Transporters in the nervous system

## Abstract

Angelman syndrome is a neurodevelopmental disorder caused by loss of function of the maternally expressed *UBE3A* gene. Treatments for the main manifestations, including cognitive dysfunction or epilepsy, are still under development. Recently, the Cl^−^ importer Na^+^-K^+^-Cl^−^ cotransporter 1 (NKCC1) and the Cl^−^ exporter K^+^-Cl^−^ cotransporter 2 (KCC2) have garnered attention as therapeutic targets for many neurological disorders. Dysregulation of neuronal intracellular Cl^−^ concentration ([Cl^−^]_i_) is generally regarded as one of the mechanisms underlying neuronal dysfunction caused by imbalanced expression of these cation-chloride cotransporters (CCCs). Here, we analyzed the regulation of [Cl^−^]_i_ and the effects of bumetanide, an NKCC1 inhibitor, in Angelman syndrome models (*Ube3a*^m−/p+^ mice). We observed increased NKCC1 expression and decreased KCC2 expression in the hippocampi of *Ube3a*^m−/p+^ mice. The average [Cl^−^]_i_ of CA1 pyramidal neurons was not significantly different but demonstrated greater variance in *Ube3a*^m−/p+^ mice. Tonic GABA_A_ receptor-mediated Cl^−^ conductance was reduced, which may have contributed to maintaining the normal average [Cl^−^]_i_. Bumetanide administration restores cognitive dysfunction in *Ube3a*^m−/p+^ mice. Seizure susceptibility was also reduced regardless of the genotype. These results suggest that an imbalanced expression of CCCs is involved in the pathophysiological mechanism of *Ube3a*^m−/p+^ mice, although the average [Cl^−^]_i_ is not altered. The blockage of NKCC1 may be a potential therapeutic strategy for patients with Angelman syndrome.

## Introduction

Angelman syndrome (AS) is a neurodevelopmental disorder caused by the loss of function of the maternally expressed gene *UBE3A*, located on chromosome 15q11–q13^1^. The primary manifestations are developmental delay, language impairment, ataxic gait, epilepsy, and characteristic behaviors, such as paroxysmal laughter^2,3^. The phenotypes, including cognitive function impairment, motor dysfunction, and abnormalities seen on electroencephalogram, are well replicated in AS model mice, which lack maternal copies of the *Ube3a* gene (*Ube3a*^m–/p+^)^4–8^. While the generation of these mouse models has contributed to remarkable progress in understanding the pathophysiological mechanisms of AS^7,9–11^, therapeutics for curing AS symptoms are still under development.

Recent evidence has suggested that the Na^+^-K^+^-Cl^+^ cotransporter 1 (NKCC1), which transports Cl^−^ into the cell, is an attractive therapeutic target for ameliorating the symptoms of a variety of neurological and psychiatric disorders, including neurodevelopmental disorders and epilepsy^12–16^. NKCC1 is widely expressed in multiple cell types, including neurons in the central nervous system (CNS), and is particularly involved in regulating the neuronal intracellular Cl^−^ concentration ([Cl^−^]_i_^17^ in cooperation with K^+^-Cl^−^ cotransporter 2 (KCC2), which facilitates Cl^−^ efflux^18^. Neuronal NKCC1 expression and function are upregulated from the prenatal to early postnatal period and gradually decrease with development. In contrast, KCC2 is dominant in mature neurons^19,20^. Various cell signaling cascades regulate changes in the expression of these cation-chloride cotransporters (CCCs) according to developmental and pathophysiological conditions, and modulate GABA-mediated signaling by regulating [Cl^−^]_i_^21,22^. A shift in the Cl^−^ equilibrium potential towards positive values accompanied by an increased NKCC1/KCC2 expression ratio have been reported in various mouse models of genetic neurodevelopmental disorders, including fragile X syndrome^12^, Rett syndrome^23^, and Down syndrome^13^, as well as in tissues derived from human patients with tuberous sclerosis^24^. Recent studies have shown that the reduction of upregulated NKCC1 expression by RNA interference successfully rescued the core symptoms of CNS disorders in mouse models of genetic diseases^25,26^. Such dysregulation of CCCs activity may be a common pathophysiological mechanism underlying genetic neurodevelopmental disorders.

Bumetanide, a loop diuretic that inhibits renal Na^+^-K^+^-Cl^+^ cotransporter 2, is also known to inhibit NKCC1. In vitro physiological experiments have shown that blocking NKCC1 with bumetanide decreases neuronal [Cl^−^]_i_, which reinforces GABA-mediated inhibitory synaptic transmission by increasing the driving force for Cl^−^^17,20^. These lines of evidence are widely regarded as indicating the pharmacological mechanisms underlying the therapeutic effects of systemic administration of bumetanide on neurological dysfunction in various rodent models of neurodevelopmental disorders^12,13^, neurodegenerative disorders^15^, and epilepsy^27^. Conversely, several researchers have argued that the effects of NKCC1 blockage by systemic administration of bumetanide cannot be simply explained by the readjustment of increased neuronal [Cl^−^]_i_, as NKCC1 is ubiquitously expressed in non-neuron brain cells, including glial cells and choroid plexus epithelial cells^16^.

Bumetanide administration in human clinical trials has yielded conflicting results. While pilot trials for autism spectrum disorder have shown that bumetanide administration can improve behavioral problems without inducing any severe side effects^28,29^, a more recent study failed to show superior effects to a placebo^30^. One of the reasons for this discrepancy may be the limited CNS penetration of bumetanide^27^. Unlike preclinical studies using rodent models, it is difficult to obtain a sufficient concentration to inhibit NKCC1 in the brain under clinical trials, whose protocol is similar to that of diuretic purposes. A novel NKCC1 inhibitor that penetrates the CNS more efficiently and is more specific to NKCC1 than bumetanide is currently under development^16,31^.

Here, we analyzed the effects of homeostatic [Cl^−^]_i_ regulation and bumetanide on neuronal dysfunction in *Ube3a*^m–/p+^ mice. Our data revealed the presence of increased NKCC1 and decreased KCC2 expression in the hippocampi of *Ube3a*^m–/p+^ mice. Although the steady-state [Cl^−^]_i_ of CA1 pyramidal neurons was not significantly different on average, it demonstrated significantly more variance in *Ube3a*^m−/p+^ mice. Chronic administration of bumetanide at doses sufficient to inhibit brain NKCC1, ameliorated novel object recognition dysfunction in *Ube3a*^m−/p+^ mice. This treatment was also effective at reducing susceptibility to pharmacologically induced seizures in both *Ube3a*^m−/p+^ mice and littermate controls. These findings suggest that blocking NKCC1 activity may be a potential therapeutic strategy for improving cognitive dysfunction and epilepsy in individuals with AS.

## Results

### CCCs expression is dysregulated in the hippocampus of ***Ube3a***^m−/p+^ mice

**Figure 1:**
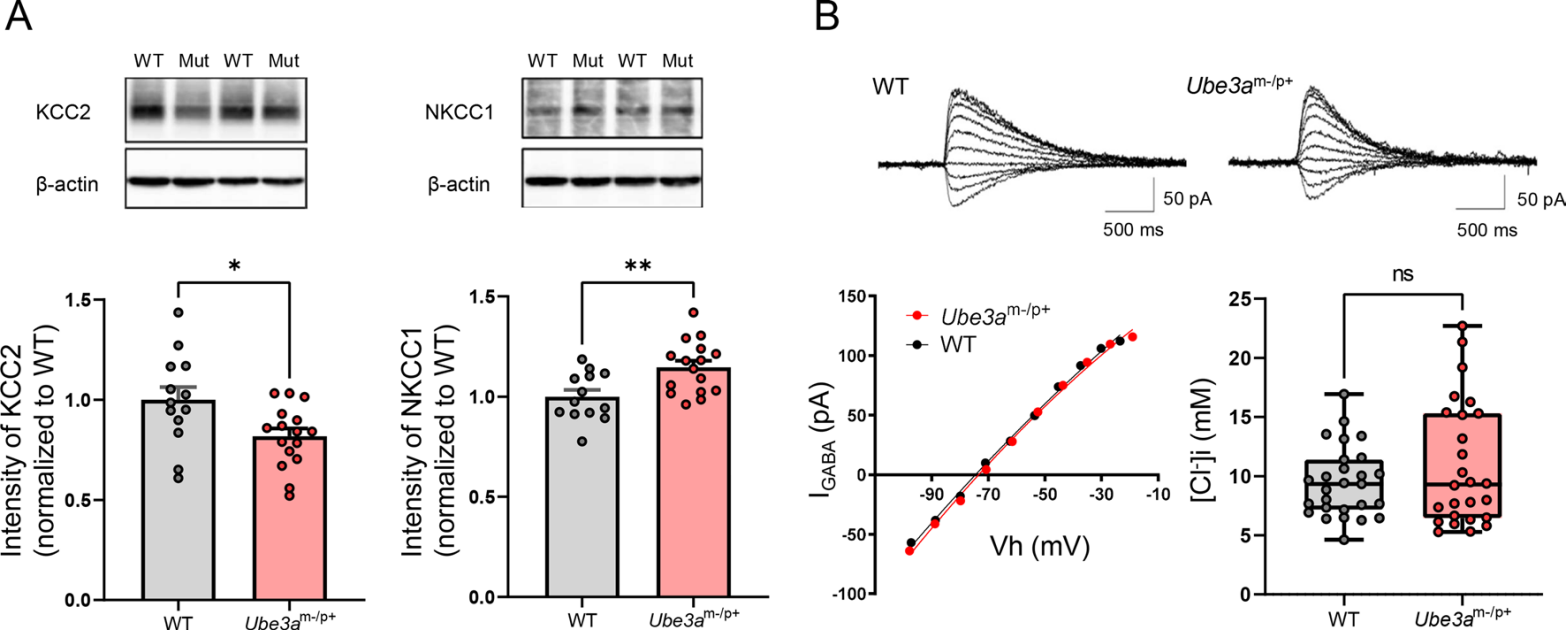
Homeostatic regulation of neuronal intracellular Cl^−^ levels in *Ube3a*^m−/p+^ mice. (**A**) Western blot analysis of the K^+^-Cl^−^ cotransporter 2 (KCC2) and Na^+^-K^+^-Cl^−^ cotransporter 1 (NKCC1) expression in hippocampus tissue lysates from wild-type (WT; n = 13) and *Ube3a*^m−/p+^ (n = 16) mice. The upper panels show KCC2 and NKCC1 immunoblots from two mice from each group. Wild type and *Ube3a*^m−/p+^ are abbreviated as “WT” and “Mut,” respectively. The original gels are shown in Supplementary information Fig. S1 and Fig. S2. The lower left and right panels show the quantification of KCC2 and NKCC1 expression. The KCC2 and NKCC1 band intensities were normalized to the β-actin band intensity. (**B**) The Upper panels show representative GABA-induced current traces at different command potentials under gramicidin-perforated patch-clamp recordings. The current–voltage relationship estimated from the peak current measured during the responses is plotted in the left lower panel. The right lower panel shows intracellular Cl^−^ concentration ([Cl^−^]_i_) of CA1 pyramidal neurons calculated from the reversal potentials of GABA-mediated currents determined in gramicidin perforated patch-clamp recordings. The lines in each box depict the lower quartile, median, and upper quartile values. The whiskers extending from each end of a box depict the minimum and maximum ranges. The [Cl^−^]_i_ values are not significantly different between WT (n = 27 cells) and *Ube3a*^m−/p+^ (n = 26 cells) groups, but are more widely distributed in *Ube3a*^m−/p+^ mice than in WT animals (*F* test for equality of variance, *p* < 0.01). Data are presented as the mean ± SEM, *p < 0.05; **p < 0.01, *ns* not significant by the unpaired *t* test.

To evaluate the homeostatic regulation of [Cl^−^]_i_ in *Ube3a*^m−/p+^ mice, we first studied the protein levels of two CCCs, the Cl^−^ importer NKCC1 and the Cl^−^ exporter KCC2, in the hippocampus of mature mice (3–5 months old). Immunoblot analyses of CCCs revealed a significant increase in NKCC1 expression in *Ube3a*^m−/p+^ mice (14% increase compared to wild-type (WT) mice, *p* = 0.0042, n = 16 for *Ube3a*^m−/p+^ mice and n = 13 for WT; Fig. 1A). Conversely, the KCC2 levels were decreased in *Ube3a*^m−/p+^ mice (18% decrease compared to those in WT mice, *p* = 0.0222; Fig. 1A). Previous studies have shown that during development, KCC2 expression begins to dominate NKCC1 expression, resulting in net Cl^−^ efflux via CCCs in mature neurons. The imbalance in NKCC1/KCC2 expression suggests impairment of this regulatory mechanism for [Cl^−^]_i_ in *Ube3a*^m−/p+^ mice.

To determine whether [Cl^−^]_i_ is indeed altered by this dysregulation of CCCs, we performed gramicidin perforated patch-clamp recordings in acute hippocampal slices prepared from mice at P24–32 days. Based on the Nernst equation, the [Cl^−^]_i_ was calculated using the measured *E*_GABA_. Contrary to the expectation from the CCC expression results, we observed no significant difference in steady-state [Cl^−^]_i_ (WT:9.5 ± 0.6 mM vs. *Ube3a*^m−/p+^:10.9 ± 1.0 mM, *p* = 0.2161; Fig. 1B) or *E*_GABA_ (WT: − 70.7 ± 1.5 mV vs. *Ube3a*^m−/p+^: − 67.9 ± 2.4 mV, *p* = 0.3321) between WT and *Ube3a*^m−/p+^ mice. Alternatively, the [Cl^−^]_i_ of *Ube3a*^m−/p+^ mice demonstrated significantly more variance than that of WT mice (test for equality of variance, *F* = 3.128, *p* < 0.0051). The resting membrane potential also showed no difference (WT: − 61.6 ± 1.0 mV vs. *Ube3a*^m−/p+^: − 61.8 ± 1.6 mV, *p* = 0.8342).

The lack of a difference in neuronal [Cl^−^]_i_ despite the increase in whole-hippocampal NKCC1 expression may imply that the upregulation of NKCC1 expression occurs predominantly in non-neuronal cells owing to its low expression in neuronal cells compared to other cell types in the brain^16^. Alternatively, the net [Cl^−^]_i_ increase due to imbalanced CCCs might be counterbalanced by deregulation of other membrane Cl^−^ transport mechanisms in *Ube3a*^m−/p+^ mice. This interpretation might be supported by the variance in [Cl^−^]_i_ values in *Ube3a*^m−p+^ mice.

### Tonic GABA_A_ receptor-mediated Cl^−^ conductance is decreased in the CA1 pyramidal neurons of ***Ube3a***^m−/p+^ mice

While [Cl^−^]_i_ modification by CCCs directly influences GABA_A_ receptor-mediated signal transmission, GABA_A_ receptor-mediated Cl^−^ conductance itself can also alter [Cl^−^]_i_^32–34,39^. To gain a deeper understanding of homeostatic [Cl^−^]_i_ regulation in *Ube3a*^m−/p+^ mice, we next evaluated the properties of GABA_A_ receptor-mediated signal transmission onto CA1 pyramidal neurons in acute slices prepared from mice at P24–32 days.

**Figure 2:**
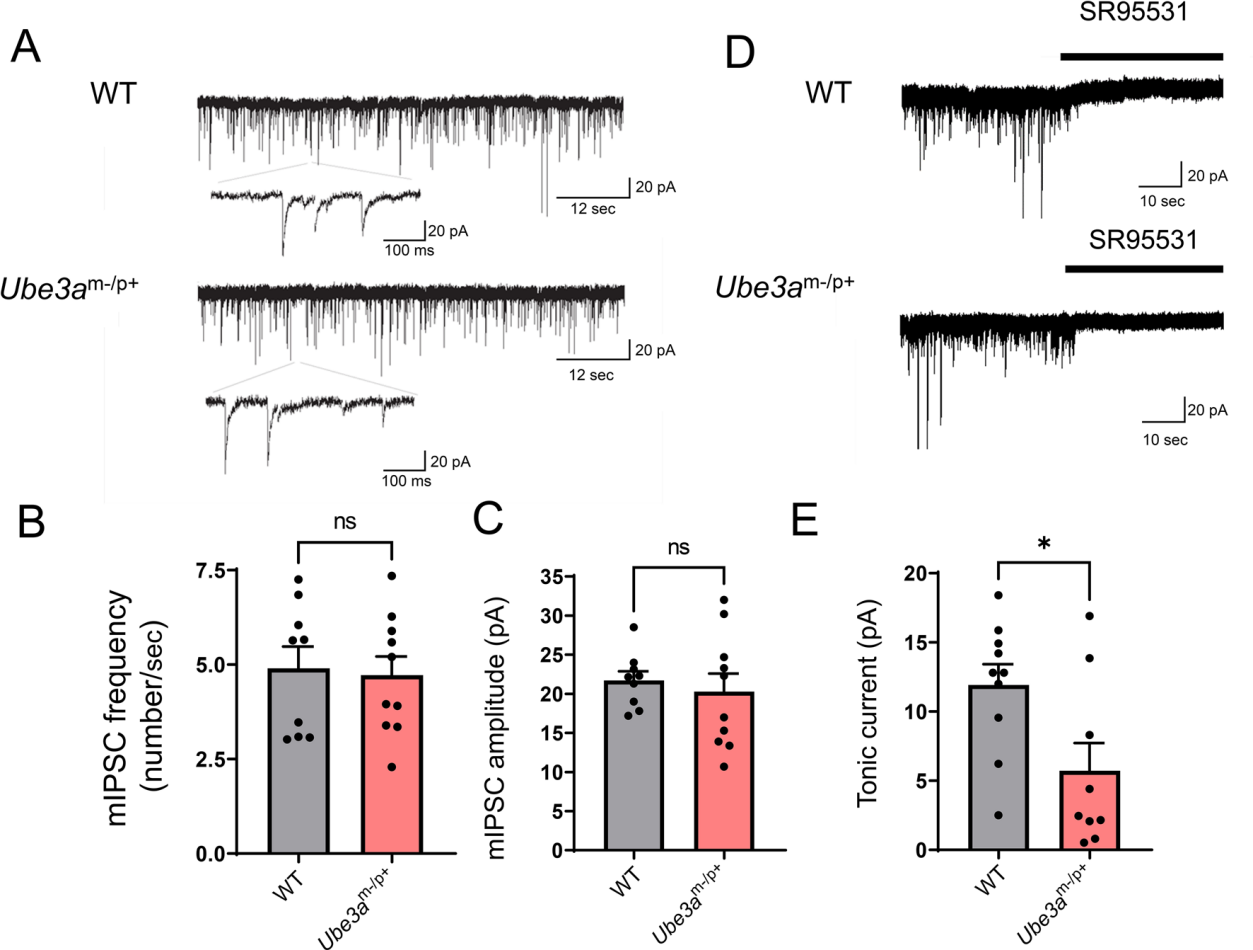
GABA_A_ receptor-mediated inhibition in CA1 pyramidal neurons of *Ube3a*^m−/p+^ mice. (**A**) Representative recording traces of miniature inhibitory postsynaptic currents (mIPSCs) in CA1 pyramidal neurons of wild-type (WT) and *Ube3a*^m-/p+^ mice. (**B**–**C**) Comparison of the mIPSC frequency (**B**) and amplitude (**C**) between WT (n = 9 cells) and *Ube3a*^m−/p+^ (n = 10 cells) groups. (**D**) Representative current traces for evaluating tonic GABA_A_ receptor-mediated inhibition in CA1 pyramidal neurons. The tonic current amplitude was determined as the baseline current shift induced by focal application of SR95531 (10 μM). € Comparison of the tonic current amplitudes in WT (n = 10 cells) and *Ube3a*^m−/p+^ (n = 9 cells) mice. The tonic form of inhibition is significantly decreased in the *Ube3a*^m−/p+^ group. Data are presented as the mean ± SEM, *p < 0.05, *ns* not significant by the unpaired *t* test.

We recorded mIPSCs using whole-cell voltage-clamp recordings in the presence of tetrodotoxin and observed no significant differences in the frequency, amplitude, or time kinetics between WT and *Ube3a*^m−/p+^ mice (Fig. 2A–C, Table 1). Short-term synaptic plasticity, evaluated by the average paired-pulse ratio of evoked inhibitory postsynaptic currents, was also unaltered in the *Ube3a*^m−/p+^ mice (Table 1). Similar results were previously reported in other brain regions, including the layer 2/3 of the visual cortex^9^ and granule cell layer of the cerebellum^7^ of young adult *Ube3a*^m−/p+^ mice. In contrast, the frequency of mEPSCs was significantly decreased in *Ube3a*^m−/p+^ mice (Table 1), in accordance with previous reports^35,36^. Our observations suggest that GABA_A_ receptor-mediated synaptic properties, including the strength and number of synapses, postsynaptic receptor properties, and presynaptic transmitter release, are maintained in *Ube3a*^m−/p+^ mice at this age.

**Table 1:** Properties of excitatory and inhibitory synapse transmission onto CA1 pyramidal neurons.

	WT	*Ube3a*^m−/p+^	*p* value
mEPSC frequency (n/min)	18.10 ± 3.30 (n = 8)	7.90 ± 1.40 (n = 8)	< 0.05
mEPSC amplitude (pA)	11.40 ± 0.63 (n = 8)	10.46 ± 0.85 (n = 8)	0.38
mEPSC rise time (ms)	3.76 ± 0.15 (n = 8)	3.90 ± 0.15 (n = 8)	0.51
mEPSC decay (ms)	4.73 ± 0.28 (n = 8)	4.66 ± 0.42 (n = 8)	0.26
PPR for evoked EPSCs	1.36 ± 0.06 (n = 12)	1.55 ± 0.09 (n = 10)	0.22
mIPSC frequency (n/s)	4.90 ± 0.58 (n = 9)	4.71 ± 0.50 (n = 10)	0.81
mIPSC amplitude (pA)	21.70 ± 1.20 (n = 9)	20.20 ± 2.30 (n = 10)	0.61
mIPSC rise time (ms)	2.69 ± 0.11 (n = 9)	2.39 ± 0.15 (n = 10)	0.13
mIPSC decay (ms)	8.83 ± 0.39 (n = 9)	7.79 ± 0.77 (n = 10)	0.26
PPR for evoked IPSCs	0.78 ± 0.03 (n = 10)	0.82 ± 0.05 (n = 10)	0.43

*mEPSC* miniature excitatory postsynaptic current, *mIPSC* miniature inhibitory postsynaptic current, *PPR* paired-pulse ratio, *WT* wild-type.

The mammalian CNS has two forms of inhibition: phasic (synaptic) inhibition and tonic inhibition. Tonic inhibition is mediated by GABA_A_ receptors expressed in the extrasynaptic membrane, which are tonically activated by ambient GABA in the extrasynaptic space^37^. We and other groups have previously shown that tonic inhibition of cerebellar granule cells is significantly decreased in *Ube3a*^m−/p+^ mice^7,38^. In accordance with these reports, tonic GABA_A_ receptor-mediated currents of CA1 pyramidal neurons were significantly reduced in *Ube3a*^m−/p+^ mice compared to those in WT animals (*p* = 0.0443, Fig. 2D, E). GABA_A_ receptor-mediated tonic Cl^−^ flux can alter [Cl^−^]_i_ and diminish the driving force for Cl^−^, which is initially determined by active Cl^−^ transport and membrane potential. Cl^−^ imaging techniques have demonstrated that blocking tonic inhibition results in a decrease in [Cl^−^]_i_ in mature cerebellar granule cells^39^. Thus, a decrease in tonic Cl^−^ conductance may be one of the mechanisms underlying the lack of significant differences in neuronal [Cl^−^]_i_ despite the imbalanced CCC expression, and could explain the variance of [Cl^−^]_i_ values in *Ube3a*^m−/p+^ mice.

### Chronic administration of bumetanide improves cognitive dysfunction in ***Ube3a***^m−/p+^ mice

The increase in NKCC1 expression suggests a therapeutic effect of bumetanide on neuronal dysfunction in *Ube3a*^m−/p+^ mice. Thus, we evaluated the effects of chronic bumetanide administration (21–28 days) on behavior, seizure susceptibility, and EEG activity in *Ube3a*^m−/p+^ mice (6–8 months old). A previous study^27^ showed that it is difficult to maintain effective bumetanide concentrations in the brain when the drug is administered only once or twice a day due to the rapid drug elimination and low brain penetration in rodents. Therefore, we utilized osmotic pumps to allow the continuous delivery of bumetanide^40^ at a flow rate adjusted to exceed the target plasma concentration, as previously shown^27^. This protocol induced substantial diuretic effects (1.5 ± 0.2 mL/24 h in WT treated with control vehicle vs. 4.0 ± 0.9 mL/24 h in WT treated with bumetanide, *p* = 0.0215, n = 8 for each group), but did not cause obvious adverse effects as shown previously^27^.

**Figure 3:**
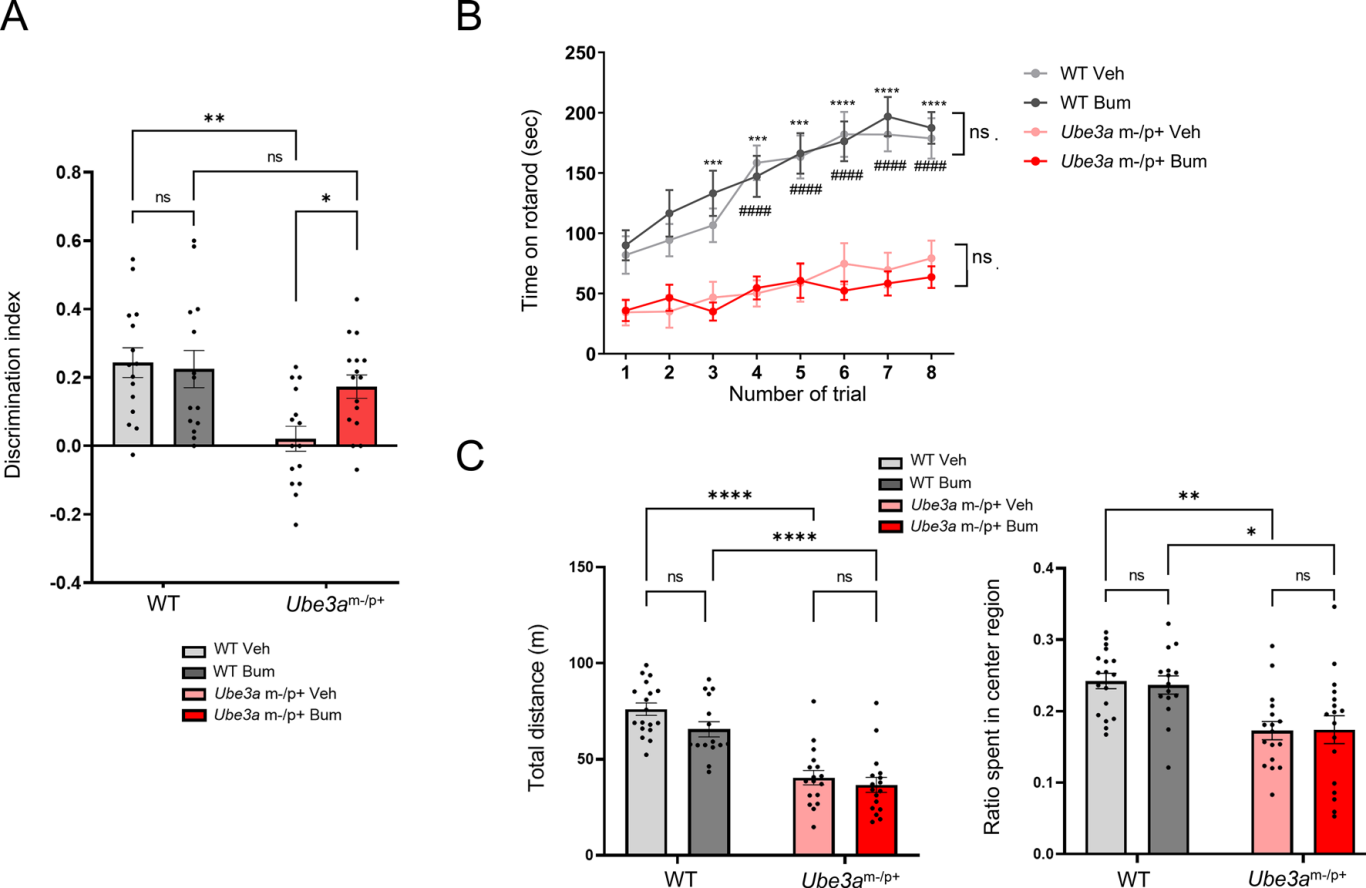
Effects of chronic bumetanide administration on behavioral test results in *Ube3a*^m−/p+^ mice. (**A)** Effects of bumetanide on the results of the novel object recognition test. *ns* not significant, *p < 0.05; **p < 0.01 by *post-hoc* analysis after two-way *ANOVA.* n = 14–16 for each group. (**B**) Effects of bumetanide on the performance of mice in the accelerating rotarod test. *ns* notsignificant between treatment with bumetanide (Bum) and vehicle control (Veh). ***p < 0.001; ****p < 0.0001 for wild-type (WT) Veh versus *Ube3a*^m−/p+^ Veh. ####: *p* < 0.0001 for WT Bum versus *Ube3a*^m−/p+^ Bum by *post-hoc* analysis after three-way repeated-measures *ANOVA.* n = 15–18 for each group. (**C**) Effects of bumetanide on the results in the open field test. The left and right panels show the total distance traveled and ratio time spent in the center region, respectively. *ns* not significant. *p < 0.05; **p < 0.01; ****p < 0.0001 by *post-hoc* analysis after two-way *ANOVA*. n = 15–18 for each group. The open field and rotarod test results are not affected by bumetanide, but bumetanide restores the low discrimination index of *Ube3a*^m−/p+^ mice in the novel object recognition test.

As a phenotype of impaired cognitive function, *Ube3a*^m−/p+^ mice showed poor novelty discrimination in the novel object recognition test (main effect of factor genotype: *F* [1,56] = 10.51, *p* = 0.0020, WT treated with control vehicle (WT Veh) vs. *Ube3a*^m−/p+^ treated with control vehicle (*Ube3a*^m−/p+^ Veh): *p* = 0.0027, Fig. 3A), consistent with a previous report^41^. We observed a significant interaction between the factors, genotype and drug in novelty discrimination capability (*F* [1,56] = 4.115, *p* = 0.0473). Subsequent *post-hoc* analysis revealed that *Ube3a*^m−/p+^ mice treated with bumetanide (*Ube3a*^m−/p+^ Bum) showed higher novelty discrimination capability than *Ube3a*^m−/p+^ Veh-treated mice (*p* = 0.0468), whereas no significant difference was detected between bumetanide-treated (WT Bum) and WT Veh (*p* = 0.7594). These results indicate that chronic administration of bumetanide effectively improved cognitive dysfunction in *Ube3a*^m−/p+^ mice.

The effect of bumetanide on motor dysfunction was evaluated using a rotarod test. As reported previously^5,7,41^, *Ube3a*^m−/p+^ mice spent a shorter time on an accelerating rotarod (main effect of factor genotype: *F* [1, 57] = 80.85, *p* < 0.0001) with a lower time increment in repetitive trials (interaction between factor genotype and time: *F* [7, 399] = 6.573, *p* < 0.0001) than WT animals. Chronic administration of bumetanide did not alter the time spent on the rotarod in either WT or *Ube3a*^m−/p+^ mice (main effect of factor drug: *F* [1, 57] = 0.02198, *p* = 0.8827; interaction between genotype and drug: *F* [1, 57] = 0.4161, *p* = 0.421; interaction among time, genotype, and drug: *F* [7, 399] = 0.6594, *p* = 0.7065; Fig. 3B).

Similar to the results of the rotarod test, treatment with bumetanide did not improve the reduced locomotor activity of *Ube3a*^m−/p+^ mice (analyzed by total distance traveled in the open field test; main effect of genotype factor: *F* [1, 57] = 80.85, *p* < 0.0001; WT Veh vs. *Ube3a*^m−/p+^ Veh: *p* < 0.0001; main effect of drug factor: *F* [1, 63] = 3.665, *p* = 0.0601; interaction between genotype and drug: *F* [1, 63] = 0.05939, *p* = 0.8244, *Ube3a*^m−/p+^ Veh vs. *Ube3a*^m−/p+^ Bum: *p* = 0.8890; Fig. 3C). *Ube3a*^m−/p+^ mice also showed reduced distance traveled in the center region relative to the total distance (main effect of factor genotype: *F* [1, 63] = 21.20, *p* < 0.0001, WT Veh vs. *Ube3a*^m−/p+^ Veh: *p* = 0.0045), a parameter commonly used to evaluate anxiety. This anxiety-like behavior could not be rescued by bumetanide (main effect of drug factor: *F* [1, 63] = 0.0255, *p* = 0.8811; interaction between genotype and drug: *F* [1, 63] = 0.05939, *p* = 0.8083, *Ube3a*^m−/p+^ Veh vs. *Ube3a*^m−/p+^ Bum: *p* = 0.9999; Fig. 3C).

### Bumetanide raises the seizure threshold in both ***Ube3a***^m−/p+^ and WT mice

**Figure 4:**
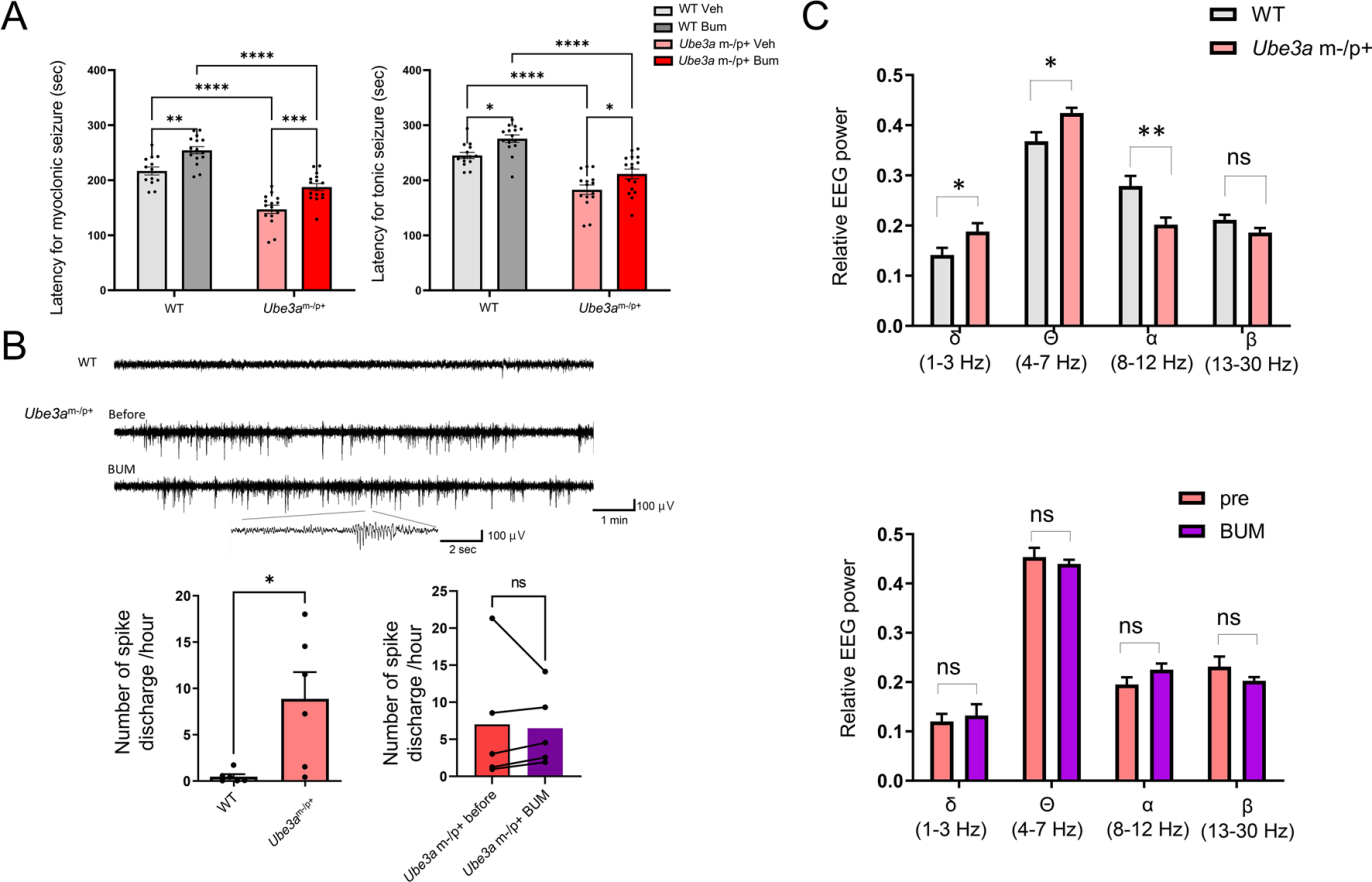
Effects of bumetanide on epileptic features of *Ube3a*^m−/p+^ mice. (**A**) Effects of bumetanide on seizure susceptibility induced by flurothyl inhalation. The left and right panels indicate the latencies of myoclonic and tonic seizures, respectively. Bumetanide increases the latency of seizure induction in both wild-type (WT) and *Ube3a*^m−/p+^ mice. *p < 0.05; **p < 0.01; ***p < 0.001; ****p < 0.0001 by *post-hoc* analysis after two-way *ANOVA*. n = 15–18 for each group. (**B**) Effects of bumetanide on epileptic discharges in *Ube3a*^m−/p+^ mice. The upper panel shows representative encephalography (EEG) traces taken during the awake state. The left lower panel demonstrates the significantly higher probability for epileptic spike discharges in *Ube3a*^m−/p+^ mice (n = 6) compared to WT animals (n = 6). *p < 0.05 by the unpaired *t* test. The right lower panel shows the probabilities of epileptic spike discharges before and after bumetanide administration in *Ube3a*^m−/p+^ mice (n = 5). *ns* not significant by the paired *t* test. (**C**) Effects of bumetanide on the relative EEG power spectrum in *Ube3a*^m−/p+^ mice. The upper panel shows the comparison of relative EEG powers in each band during the awake state between WT (n = 11) and *Ube3a*^m−/p+^ (n = 11) groups. *p < 0.05; **p < 0.01 by the unpaired *t* test. The lower panel shows the relative EEG powers in each frequency band before and after bumetanide administration in *Ube3a*^m−/p+^ mice (n = 5). *ns* not significant by the paired *t* test. Bumetanide does not affect the EEG abnormalities of *Ube3a*^m−/p+^ mice.

Most previous studies have reported that seizure susceptibility, as evaluated by an acute seizure induction paradigm, is comparable between WT and *Ube3a*^m−/p+^ of the C57BL/6 strain at a young adult age^4,41^. We recently reported that, in middle-aged mice instead of young adult C57BL/6 mice, the seizure susceptibility induced by flurothyl inhalation was significantly higher in *Ube3a*^m−/p+^ mice than in WT animals^8^. This is in accordance with the clinical observation that epilepsy symptoms become more pronounced with age after adolescence in patients with AS^42,43^. Thus, we evaluated the effects of bumetanide on seizure susceptibility induced by flurothyl inhalation in middle-aged *Ube3a*^m−/p+^ mice (6–12 months old). As shown previously, the latencies of myoclonic and tonic seizures were both significantly shorter in *Ube3a*^m−/p+^ mice than in WT mice (myoclonic seizure: main effect of genotype: *F* [1, 56] = 99.18, *p* < 0.0001, WT Veh vs. *Ube3a*^m−/p+^ Veh: *p* < 0.0001). Chronic administration of bumetanide significantly lengthened the latency in both WT and *Ube3a*^m−/p+^ mice (myoclonic seizure: main effect of drug: *F* [1, 56] = 99.18, *p* < 0.0001; WT Veh vs. WT Bum, *p* = 0.002; *Ube3a*^m−/p+^ Veh vs. *Ube3a*^m−/p+^ Bum: *p* = 0.0005; tonic seizure: main effect of drug: *F* [1, 56] = 14.88, *p* = 0.0003; WT Veh vs. WT Bum: *p* = 0.0380, *Ube3a*^m−/p+^ Veh vs. *Ube3a*^m−/p+^ Bum: *p* = 0.0446; Fig. 4A). No significant interaction was observed between genotype and drug, suggesting that flurothyl-induced seizure activity may involve NKCC1 activation, regardless of genotype.

Next, we evaluated the effects of bumetanide on the spontaneous EEG activity in *Ube3a*^m−/p+^ mice. Replicating the EEG characteristics of human individuals with AS, long-term subdural EEG recordings indicated that *Ube3a*^m−/p+^ mice showed a higher probability of epileptic discharges (WT vs. *Ube3a*^m−/p+^: *p* = 0.0160; Fig. 4B) and higher power in low-frequency bands during the awake state (WT vs. *Ube3a*^m−/p+^, δ band: *p* = 0.040, θ band: *p* = 0.0153, α band: *p* = 0.0058; Fig. 4C), consistent with previous reports^5,41,44^. The frequency of epileptic discharges and power in low-frequency bands were not altered by chronic bumetanide administration in *Ube3a*^m−/p+^ mice (*Ube3a*^m−/p+^ before Bum vs. *Ube3a*^m−/p+^ after Bum; δ band: *p* = 0.770; θ band: *p* = 0.4572; α band: *p* = 0.1279, β band: *p* = 0.3090; Fig. 4C). These results suggest that imbalanced CCC expression is not the sole cause of EEG abnormalities in *Ube3a*^m−/p+^ mice.

## Discussion

*UBE3A*, the causal gene of AS, encodes the E6AP (UBE3A) protein, an E3 ubiquitin ligase in the protein-proteasome pathway. While several neuronal proteins have been identified as specific substrates for ubiquitination by UBE3A^45^, accumulating evidence has indicated that several neuronal cell signaling pathways are indirectly altered by UBE3A loss of function^11^. This implies that an enormous number of proteins can be dysregulated in AS, and that AS pathophysiology may therefore differ according to phenotype^10^. Identifying pathogenic proteins that enable the pharmacological manipulation of dysfunction is important for the evaluation of therapeutic strategies in AS. Therefore, we focused on CCCs and found that their expression was aberrantly dysregulated in *Ube3a*^m−/p+^ mice. Additionally, chronic administration of bumetanide ameliorated cognitive dysfunction in *Ube3a*^m−/p+^ mice, suggesting that imbalanced expression of CCCs contributes to the phenotype of cognitive dysfunction in AS. Although there is no evidence showing a direct interaction between UBE3A and CCCs, these results imply that UBE3A can affect portions of the cell signaling pathways that regulate CCCs alterations in response to developmental changes or pathophysiological conditions^22^. Brain-derived neurotrophic factor (BDNF)-tropomyosin receptor kinase B (TrkB) signaling, which facilitates CCCs alternation during development^46^, may be a candidate because the impairment of TrkB signaling has been shown in *Ube3a*^m−/p+^ mice^47^. However, details of the interaction between UBE3A and BDNF-TrkB signaling are not fully understood. Thus, further analysis is required to clarify the mechanism underlying dysregulation of CCCs expression in *Ube3a*^m−/p+^ mice.

Similar ameliorating effects of bumetanide on cognitive impairment have been reported in other mouse models of CNS diseases, including Down syndrome^13^ and Huntington’s disease^15^. In contrast to prior studies that demonstrated an increased mean [Cl^−^]_i_ accompanied by increased NKCC1 expression, the [Cl^−^]_i_ value did not differ between WT and *Ube3a*^m−/p+^. While electroneutral CCCs are the primary determinants of neuronal [Cl^−^]_i_, passive Cl^−^ conductance via Cl^−^ channels and anion exchangers has been proposed as an additional regulator of [Cl^−^]_i_^33^. In particular, previous studies have indicated that both synaptic and tonic GABA_A_ receptor conductance can alter [Cl^−^]_i_ under physiological conditions^32–34,39^. In the present study, we showed that tonic GABA_A_ receptor-mediated conductance decreased in CA1 pyramidal neurons. Previously, we reported an increase in GABA transporter 1 expression and subsequent reduction in ambient GABA as a mechanism of decreased tonic inhibition in the cerebellum of *Ube3a*^m−/p+^ mice^7^. Because this GABA transporter is also expressed in the hippocampus, a similar mechanism may be responsible for decreased tonic inhibition in the hippocampus. Given the variability of [Cl^−^]_i_ in neurons with inward- or outward-directed driving forces for Cl^−^, the decrease in passive tonic Cl^−^ influx or efflux, respectively, via extrasynaptic GABA_A_ receptors may stabilize the [Cl^−^]_i_ generated by the imbalance in CCCs expression. This may explain the comparable average [Cl^−^]_i_, as well as its increased variance, in *Ube3a*^m−/p+^ mice. Conversely, the imbalanced CCCs expression may be subsequent to the reduced Cl^−^ loading, due to decreased tonic Cl^−^ conductance^33^. Such a counterbalancing mechanism is not fully understood and is of future research interest. The variance in the [Cl^−^]_i_ distribution can be reduced by bumetanide because its effect on decreasing [Cl^−^]_i_ is more pronounced in individual neurons with a higher initial [Cl^−^]_i_^48^. Thus, our results imply that a higher steady-state [Cl^−^]_i_ in a part of the neuronal population can cause cognitive impairment even if the average [Cl^−^]_i_ is not different. To prove this speculation, [Cl^−^]_i_ and its pharmacological changes should be evaluated in a much larger number of neurons using the Cl^−^ imaging technique in future research.

To discuss the relationship between the results of western blotting for CCCs and neuronal [Cl^−^]_i_, we need to consider the distribution of NKCC1 expression in the brain. NKCC1 is demonstrably expressed in a variety of cells in the brain including glial cells, neurons and cells outside the parenchyma^49^. However, its localization in the forebrain is still under debate as the results of immunohistochemistry have been inconsistent across studies^50^. Some research groups argue that it is more dominant in non-neuronal cells than in neurons and that the mechanism underlying the therapeutic effects of bumetanide is therefore more complicated^16^. In this case, the imbalance in neuronal CCC expression would be milder than indicated by the results of western blotting, which may explain the lack of significant difference in [Cl^−^]_i_. The controversy may arise from the lack of reliable NKCC1 antibodies for immunohistochemistry^50^. A feature study investigating the distribution of increased NKCC1 levels using a novel NKCC1 antibody is required to elucidate the mechanism underlying the therapeutic effects of bumetanide in *Ube3a*^m−/p+^ mice.

Although bumetanide was also effective in increasing the seizure threshold in *Ube3a*^m−/p+^ mice, its efficacy in these mice was comparable to that in WT mice, indicating that flurothyl-induced seizure activity may involve NKCC1 activation, regardless of genotype. In contrast, EEG abnormalities, including epileptic spike discharges and higher power in the lower frequency bands, were not improved by bumetanide. A previous study in an experimental epilepsy model revealed the antiepileptic effects of low-dose bumetanide, whose concentration in the brain was incompatible with NKCC1 inhibition^51^, suggesting that target(s) other than NKCC1 may contribute to the antiepileptic effect of bumetanide. Further investigations are required to clarify the mechanisms underlying the bumetanide-induced reduction in seizure susceptibility.

In contrast to cognitive dysfunction, motor dysfunction and EEG abnormalities of *Ube3a*^m−/p+^ were not affected by the application of bumetanide. This discrepancy may be correlated with previous findings that NKCC1 distribution differs by region at the messenger RNA level^52^. These discrepant effects of bumetanide among symptoms are in line with the speculation that AS pathophysiological mechanisms induced by UBE3A deficiency differ according to the phenotype^10^. To date, the dysregulation of a variety of cation ion channels or transporters including voltage-dependent big potassium (BK) channels^53^, the Ca^2+^-activated small conductance potassium channel (SK2)^54^, Na/K-ATPase (α1-NaKA) and the Na^+^ channel (Nav1.6)^55^ have been identified as pathophysiological mechanisms in AS models. Because CCCs and these cation channel/transporters can functionally interact with each other, the therapeutic effects of CCCs inhibition may be affected by the diverse pathophysiology, which may also explain the inconsistent results of bumetanide. Further research investigating the crosstalk of multiple pathophysiology may contribute to a deeper understanding of the mechanisms underlying a variety of neuronal dysfunctions in AS.

In this study, we provide evidence that bumetanide alleviates the symptoms of AS. Off-label use of bumetanide has been tested in clinical trials in a variety of common CNS diseases, including epilepsy, ASD, tuberous sclerosis, and schizophrenia, and its efficacy is still controversial^30^. One of the main limitations of bumetanide is its low brain penetrance across the blood–brain barrier^27^. Clinical doses of bumetanide approved as a diuretic are insufficient to inhibit brain NKCC1. To overcome this issue, prodrugs of bumetanide, which show more efficient brain permeability and/or NKCC1 selectivity, are currently under development^16,31^. The efficacy of bumetanide was not marked in this study, despite its high-dose administration. Due to compliance and adverse effects, our results are difficult to translate into clinical applications directly. Nevertheless, our findings highlight the pathophysiological involvement of imbalanced CCCs expression in AS and may lead to novel therapeutic strategies for ameliorating the symptoms of AS.

## Methods

### Animals

C57BL/6 mice were used to generate mice carrying a *Ube3a* mutation at Nagasaki University^5^ and were shipped to Hokkaido University Graduate School of Medicine or Hamamatsu University School of Medicine for use in all experimental procedures. *Ube3a*^m−/p+^ mice were obtained by crossing a heterogeneous female mouse lacking paternal *Ube3a* with a WT male mouse. Genotyping was performed using polymerase chain reaction of mouse tail DNA, as described previously^5^. After weaning, 3–5 mice were housed in ventilated cages with water and feed provided ad libitum. Mice were housed under a 12-h light/ 12-h dark cycle (lights on at 7:00 a.m.), with temperature and humidity maintained at 23–25 °C and 45–55%, respectively. All experimental procedures were approved by the Institutional Animal Care and Use Committee of the Hamamatsu University School of Medicine and Hokkaido University, and followed the National Institutes of Health guidelines and ARRIVE (Animal Research: Reporting of In Vivo Experiments) guidelines for the care and use of laboratory animals.

### Immunoblotting

The hippocampal regions were dissected from the collected brains and homogenized in ice-cold lysis buffer (50 mM Tris–HCl, 150 mM NaCl, 5 mM ethylenediaminetetraacetic acid, and 1% Triton X-100) containing protease inhibitors (Roche, #1697498). The samples were centrifuged for 10 min at 12,000×*g* at 4 °C, and the supernatants were mixed with Laemmli sample buffer and heated at 100 °C for 5 min. The protein concentrations of the samples were determined using the Bio-Rad DC protein assay (Bio-Rad). Equal amounts of proteins were subjected to sodium dodecyl sulfate–polyacrylamide gel electrophoresis (7.5% acrylamide gel) and transferred to polyvinylidene fluoride membranes. The membranes were then blocked in 1% bovine serum albumin and incubated overnight with antibodies against the target proteins at 4 °C. The blots were then incubated with a horseradish peroxidase-conjugated secondary antibody (GE Healthcare) for 1 h at room temperature (23–25 °C). Bands were visualized with ECL prime or ECL select western blot detection reagent (Cytiva) and imaged using a ChemiDoc MP imaging system (Bio-Rad). Quantification was performed using Image Lab software (version 6.0, Bio-Rad). The KCC2 and NKCC1 band intensities were normalized to β-actin band intensity. Data were obtained from a separate set of mice and stored for statistical analysis. The following primary antibodies were used for the target proteins. KCC2: Millipore, #07-432 (1:1000); NKCC1: Millipore, #MABS1237 (Millipore Sigma, Burlington, MA, USA) (1:1000) for the first cohort or Developmental Studies Hybridoma Bank, University of Iowa, T4 (University of Iowa, Iowa City, IA, USA) (1:2000) for the second cohort; and β-actin (1:5000; Sigma-Aldrich, #A5441) (Sigma-Aldrich, St. Louis, MS, USA). Both antibodies against NKCC1 were T4 monoclonal antibodies and were not NKCC isoform-specific. However, NKCC2 transcript and proteins are not present in the brain^56^ and their specificity for NKCC1 in the adult hippocampus has been validated by the detection of no signals on western blotting obtained from NKCC1 null mutant mice^57^.

### Electrophysiology

Experiments were performed on acute hippocampal slices prepared from P25–28 *Ube3a*^m−/p+^ or WT littermate mice. Both male and female mice were included in the electrophysiological experiments. We recorded each genotype on consecutive days, whenever possible. Mice were killed by decapitation under deep anesthesia using halothane or isoflurane, and brain coronal slices containing the hippocampus (350 μm thick) were cut on a microslicer (VT-1000S, Leica Microsystems; or VF-300-0Z, Precisionary) in ice-cold modified artificial cerebrospinal fluid (ACSF) containing (in mM): 220 sucrose, 2.5 KCl, 1.25 NaH_2_PO_4_, 12.0 Mg_2_SO_4_, 0.5 CaCl_2_, 26.0 NaHCO_3_, and 30.0 glucose, pH 7.4 when gassed with 95% O_2_/5% CO_2_. The slices were kept in standard ACSF solution consisting of (in mM) 126 NaCl, 2.5 KCl, 1.25 NaH_2_PO_4_, 2.0 MgSO_4_, 2.0 CaCl_2_, 26.0 NaHCO_3_, and 20.0 glucose, pH 7.4 when gassed with 95% O_2_/5% CO_2_, at room temperature for over 1 h before experiments^58^.

Slices were then transferred to a recording chamber on the stage of a microscope (BX61, Olympus, or Axioskop2, Zeiss) and continuously perfused with oxygenated ACSF at a flow rate of 2 ml/min at 30 °C. CA1 pyramidal neurons were visually identified on a monitor using a 40 × water immersion objective lens with an infrared differential interference contrast filter. The patch electrodes were pulled from borosilicate capillary tubing with a 1.5 mm diameter (GD-1.5; Narishige) with a horizontal puller P-97 (Sutter Instruments). The electrode resistance ranged from 4 to 6 MΩ for conventional whole-cell patch-clamp recordings and from 3 to 5 MΩ for gramicidin-perforated patch-clamp recordings. Signals were recorded using a MultiClamp 700 B amplifier or Axopatch 200 B (Molecular Devices), low-pass filtered at 2 kHz, and digitized at 6–10 kHz using a Digidata 1332A data acquisition system (Molecular Devices).

Gramicidin perforated patch-clamp recordings were performed as previously described^17^. The pipette solution contained (150 mM): 150 KCl and 10 mM HEPES (pH 7.3, KOH). Gramicidin was dissolved in DMSO (10 mg/ml) and then diluted in the pipette-filling solution to a final concentration of 5–10 μg/ml immediately prior to the experiments. The cells were voltage clamped and stepped into various test potentials. GABA (50 μM) was applied for 10 ms through a patch pipette to the soma of the recorded neuron at each membrane potential. To obtain *I–V* curves from gramicidin recordings, the membrane potential values were corrected for the voltage drop across the series resistance: *V*_corr_ = *V*_com_ − *I*_clamp_ × R_s_, where *V*_com_ is the command potential, *I*_clamp_ is the clamp current, and R_s_ is the series resistance^17^. To determine the reversal potential for the GABA-induced current (*E*_GABA_), these values were plotted as a function of the series resistance-corrected membrane potential. [Cl^−^]_i_ was calculated from the determined *E*_GABA_ according to the Nernst equation.

To evaluate the GABA_A_ receptor-mediated currents, whole-cell voltage-clamp recordings were performed under the presence of 6-cyano-7-nitroquinoxaline-2, 3-dione (CNQX; 20 µM), D-(-)-2-Amino-5-phosphonopentanoic acid (D-AP5; 50 µM), and CGP55845 (3 µM). Recorded neurons were voltage-clamped at a holding potential of − 60 mV using a pipette solution consisting of (in mM) 130 CsCl, 1 mM CaCl_2_, 2 MgCl_2_,10 HEPES–NaOH, 0.5 EGTA-KOH, 1.5 Mg-ATP, 0.5 mM Na_2_-GTP, and 2.5 QX314 (pH 7.3). Tonic GABA currents were evaluated as the difference in the mean baseline current devoid of synaptic events (total 3 s) during and before the application of SR95531 (10 µM)^7^. To evaluate excitatory postsynaptic currents, whole-cell voltage-clamp recordings were performed in the presence of SR95531 (10 µM) and CGP55845 (3 µM). The voltage was clamped at − 60 mV with the pipette solution consisting of (in mM): 150 K-CH_3_SO_3_, 5 KCl, 3 MgCl_2_, 10 HEPES–NaOH, 0.5 EGTA-KOH, 3 Mg-ATP, 0.4 Na_2_-GTP (pH 7.3). Resting membrane potentials were recorded using the same pipette solution. The reported values were corrected for liquid junction potentials of + 10.5 mV.

For recordings of miniature inhibitory currents (mIPSCs) or excitatory postsynaptic currents (mEPSCs), tetrodotoxin (1 µM) was additionally applied to the perfusion solution. Individual mIPSCs and mEPSCs were visually identified from 5-min current traces to analyze their frequency, peak amplitude, 10–90% rise time, and decay time using Mini Analysis (Synaptosoft, NJ). The data obtained from each event were averaged. For the analysis of paired-pulse ratios, two consecutive inhibitory or excitatory postsynaptic currents were evoked by electrical stimulation (interval, 50 ms; duration, 200 µs; intensity, 100–400 pA) using a monopolar glass pipette filled with ACSF. The ratios of the peak current amplitudes between the second and first evoked postsynaptic currents were determined.

### Long-term administration of bumetanide

We used micro-osmotic pumps (Model, 2004, Alzet, USA) to allow continuous administration of bumetanide^40^. Pumps were filled with 25.6 mg bumetanide in 200 μl 70% PEG/30% DMSO to deliver approximately 0.8 mg kg^−1^ h^−1^ for up to 28 days and subcutaneously implanted under anesthesia. This infusion rate allows for the achievement of sufficient plasma concentration for inhibiting NKCC1 in the rodent brain without any adverse effect^27^. All subsequent experiments were performed between 21 and 28 days after implantation. The diuretic effects of this protocol were also evaluated from 24-h urine volume in WT mice by holding a mouse in a metabolic cage for 24 h.

### Behavioral analysis

All behavioral analyses were conducted during the light cycle using 6-to 8-month-old male mice. Prior to each test, mice were habituated to the testing room for at least 60 min. For the novel object recognition and open field tests, a video tracking system (ANY-maze; Stoelting Co., USA) was used to capture the procedures.

The task procedure of the novel object recognition test consists of three phases: habituation, familiarization, and test^59^. Each mouse was allowed to explore the empty arena (40 × 40 × 40 cm) freely for 5 min, which was followed by exploration of two identical sample objects for 5 min. After a retention interval of 30 min, the mice were returned to the arena for 5 min during the test phase. In this phase, one of the two samples is replaced with a novel object. The number of times a mouse showed exploratory behavior (direct contact or sniffing toward an object within less than 2 cm of the object) was manually counted offline by an examiner who was blinded to the subjects. The discrimination index (DI) was calculated using the following equation:

$${\text{DI }} = \, \left( {{\text{N}}_{{\text{N}}} - {\text{ N}}_{{\text{F}}} } \right) \, / \, \left( {{\text{N}}_{{\text{N}}} + {\text{ N}}_{{\text{F}}} } \right),$$

where N_N_ and N_F_ represent the number of exploration times for the novel and familiar objects, respectively.

Motor function was analyzed using an accelerating rotarod (4–40 rpm for 5 min; model MK-670, Muromachi, Japan). The mice were trained to stay on the rod at a constant speed (5 rpm for 5 min) prior to data acquisition. Four trials per day were conducted for two consecutive days at 30 min interval. The time spent on the rotarod or the time until the mouse made three consecutive rotations on the rotarod was used in the subsequent statistical analysis.

For the open field test, each mouse was placed in the center of an empty arena (40 × 40 × 40 cm) and allowed to explore freely for 30 min. We analyzed the total distance traveled as well as the relative distance traveled in the center region (25 cm × 25 cm in the middle of the arena) in relation to the total distance traveled.

### Flurothyl inhalation-induced seizures

Seizure susceptibility of 6- to 8-month-old male mice was evaluated by an acute seizure induction paradigm using flurothyl inhalation, as reported previously^8^. Mice were placed in an airtight acrylic cylinder chamber (diameter, 14 cm; height, 20 cm; Asone 1-073-01) 1 min before starting flurothyl administration. A 10% fluorothyl (bis [2,2,2-trifluoroethyl] ether; Sigma-Aldrich) solution was dispensed with 95% ethanol and infused into a gauze pad (3 × 3 cm) at a flow rate of 200 µL/min using a syringe pump suspended 5 cm below the ceiling. We recorded the latency from the beginning of administration to the first presentation of (1) a myoclonic seizure: a sudden, brief muscle contraction of the neck and body, and (2) tonic seizures: sustained loss of posture control (> 2 s) accompanied by trunk rigidity^60^. The experiment was performed in a ventilated safety cabinet to avoid exposing the examiner to a gas containing fluorothyl.

### Electroencephalography (EEG) recording and analysis

Cortical EEGs were recorded using stainless steel screw electrodes (Plastic One, USA) in 6–9-month-old male mice. Cortical recording electrodes (A/P: − 3.0 mm, ML: ± 3.0 mm, relative to bregma), a reference electrode over the cerebellum, and a grand electrode anterior to bregma were implanted subdurally under anesthesia with isoflurane. All electrodes were inserted into a 6-channel pedestal and connected to a commutator for recording. Mice were allowed at least 7 days of recovery from surgery before recording.

Simultaneous video-EEG recording was performed for 24 h. EEG signals were amplified by a differential AC amplifier (Model 1700, A-M Systems, USA), bandpass-filtered between 0.1 Hz and 500 kHz, and digitized at 2000 Hz (MP170 and AcqKnowlegde software; Biopack Systems, USA) for storage on a PC. EEG spikes were automatically detected by threshold-based event detection using the Clampfit 10 software (Molecular Devices, USA). The threshold amplitude was set to four times the standard deviation of the baseline EEG activity during non-REM sleep. Waveforms over 200 ms in duration were rejected as artifacts. Events including three consecutive spike trains within a 200-ms interval were counted as epileptic discharges.

Spectral analysis was performed using Darbeliai, a plug-in of EEGLAB^61^ running MATLAB (MathWorks, Inc., USA). After visually inspecting the EEG traces and excluding artifacts, 10 EEG epochs (duration 9 s) during the awake state were collected for power spectrum analysis. We calculated the relative EEG powers of the δ (1–4 Hz), θ (4–9 Hz), α (9–13 Hz), and β (13–30 Hz) frequency bands for each epoch. The EEG power of each mouse was determined by averaging over 10 epochs.

### Statistical analysis

Differences in immunoblotting, patch-clamp recording, and EEG data between genotypes were determined using an unpaired *t *test. Welch’s correction was applied when the *F-test* result for the comparison of variance was significant. Alterations in EEG data before and after bumetanide application in *Ube3a*^m−/p+^ mice were determined using a paired *t *test. The effects of bumetanide on behavioral analyses and seizure susceptibility were analyzed using two-way analysis of variance (*ANOVA*) (for all except the rotarod test) or three-way repeated-measures *ANOVA* (for the rotarod test) followed by Tukey’s *post-hoc* analysis. All statistical analyses were performed using Prism 9 (GraphPad Software), and statistical significance was set at *P* < 0.05.

## Supplementary Information

Supplementary Information.

## Data Availability

The datasets generated and/or analyzed during the current study are available in the zenodo repository, https://doi.org/10.5281/zenodo.7186642.
