# Supplementary material for: Imbalanced expression of cation-chloride cotransporters as a potential therapeutic target in an Angelman syndrome mouse model
Source: Sci Rep. 2023 Apr 17;13:5685. doi: 10.1038/s41598-023-32376-z (PMC10110603; doi:10.1038/s41598-023-32376-z)
Supplement: Supplementary file 1 — Supplementary Information. [file 41598_2023_32376_MOESM1_ESM.docx]

# **Supplementary information**

**Imbalanced expression of cation-chloride cotransporters as a potential therapeutic target in an Angelman Syndrome mouse model**

Kiyoshi Egawa^a,†,*^, Miho Watanabe^b, †^, Hideaki Shiraishi^a^, Daisuke Sato^a^, Yukitoshi Takahashi^c^, Saori Nishio^d^ , Atsuo Fukuda^b^

^a^ Department of Pediatrics, Hokkaido University Graduate School of Medicine, Kita 15, Nishi 7, Kita-ku, Sapporo,060-8638, Japan

^b^ Department of Neurophysiology, Hamamatsu University School of Medicine 1-20-1 Handayama, Higashi-ku, Hamamatsu city, Shizuoka, 431-3192, Japan

^c^ Department of Clinical Research, National Epilepsy Center, NHO, Shizuoka Institute of Epilepsy and Neurological Disorders, Urushiyama 886, Aoi-ku, Shizuoka 420-8688, Japan

^d^  Department of Rheumatology, Endocrinology, and Nephrology, Hokkaido University Graduate School of Medicine, Kita 15, Nishi 7, Kita-ku, Sapporo,060-8638, Japan

^†^: These authors contributed equally. Kiyoshi Egawa, MD, PhD

^*^: To whom correspondence should be addressed.

Department of Pediatrics, Hokkaido University Graduate School of Medicine

North 15, West 7, Kita-ku, Sapporo 060-8638, Japan.

Tel: +81-11-706-5954

Email: egakiyo@huhp.hokudai.ac.jp


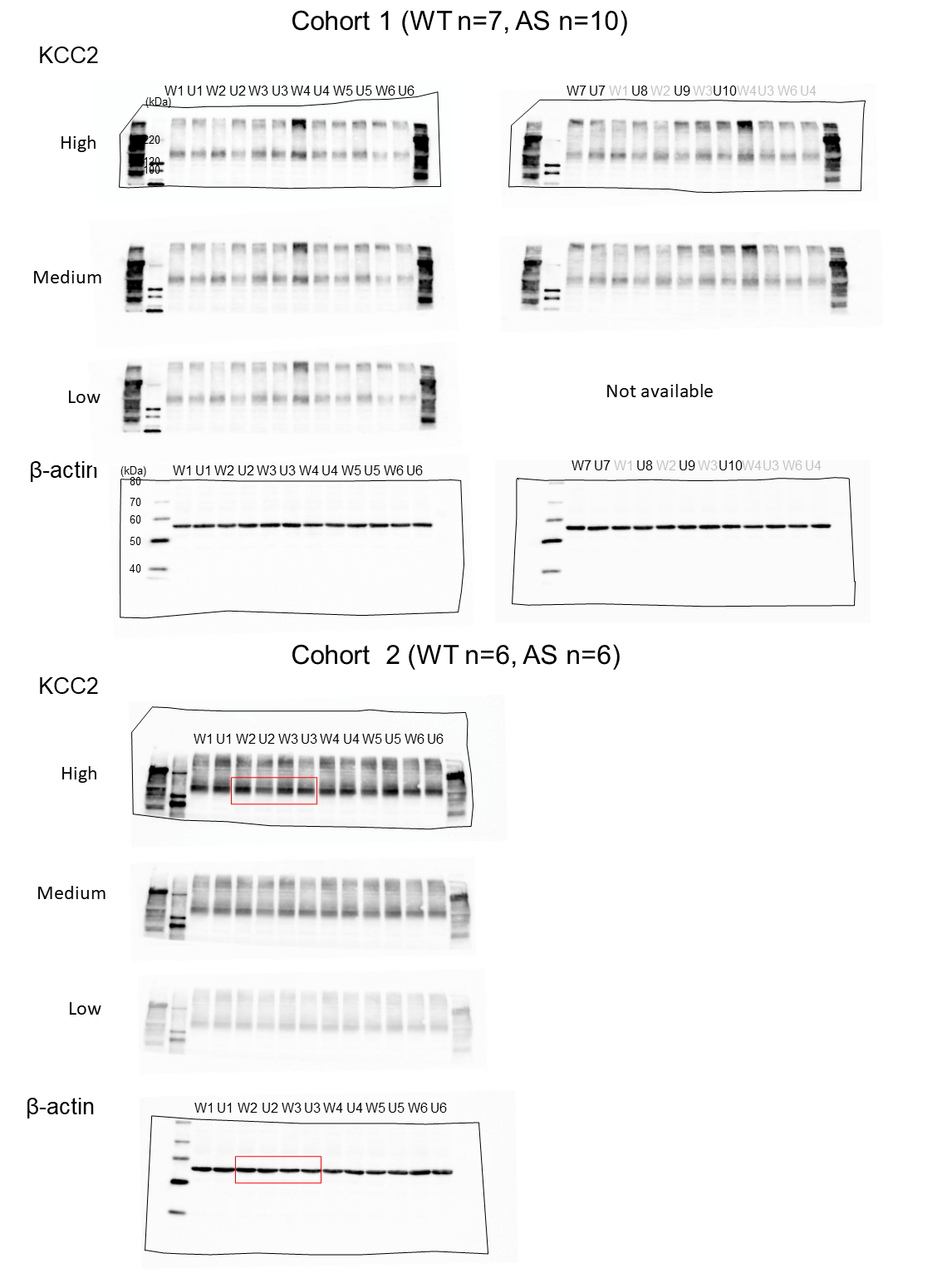


**Fig.S1. Western blot images of KCC2.** The original gels for KCC2 western blotting shown with multiple exposures (high, medium, low). The borders of the blot are indicated by a black line to contrast with the background. The red frames indicate the bands that were trimmed in Fig 1A. Wild type and *Ube3a*^m-/p+^ are abbreviated as “W” and “U,” respectively. Samples indicated by the gray font are duplicate of those indicated by the black font and were not included in the quantitative analysis


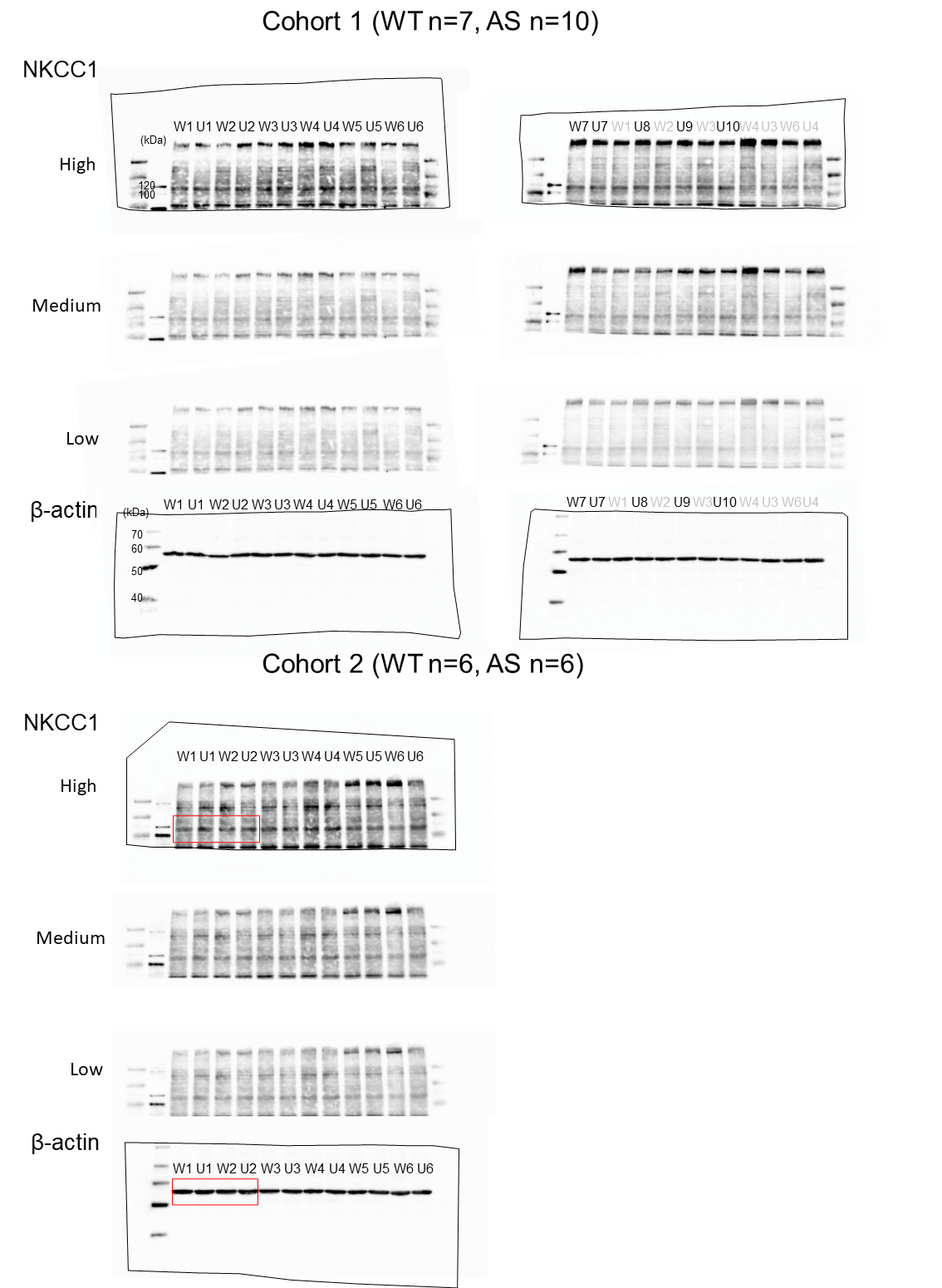


**Fig.S21 Western blot images of NKCC1.** The original gels for NKCC1 western blotting shown with multiple exposures (high, medium, low). The borders of the blot are indicated by a black line to contrast with the background. The red frames indicate the bands that were trimmed in Fig 1A. Wild type and *Ube3a*^m-/p+^ are abbreviated as “W” and “U,” respectively. Samples indicated by the gray font are duplicate of those indicated by the black font and were not included in the quantitative analysis
